# Stem Cell Quandary Quashes Research, Smithsonian Secretary Backs Down from CRC Closure - and Welcome Back to Star Wars

**DOI:** 10.1100/tsw.2001.51

**Published:** 2001-05-11

**Authors:** Shauna M. Haley

## Abstract

Nature leads this week with a story about how questions surrounding stem cell research have thrown a funding agency and the German government into the boxing ring. The contested overhaul of the U.S. Smithsonian Institution tops the news this week in Science.

The major funding agency for German science, the Deutsche Forschungsgemeinschaft (DFG), has changed its position on embryonic human stem cell research, reports *Nature*. The agency, which in 1999 released a statement discouraging scientists from working with the disputed cells, has now said that it will back embryonic stem cell investigations. But the German research ministry (BMBF) has balked at the DFG's new guidelines, calling for a pause on all funding for individual projects while the government evaluates the "ethical and moral issues" of embryonic stem cell research, says *Nature*. The DFG has also called for a revision to the government's laws regarding embryonic stem cell use in the country – to permit scientists to maintain cell lines imported from other nations. *Science* also reports on these developments in its fourth spot. Both journals note that the DFG, while having legislative distinction as an independent agency, derives about half of its monies from government sources and half of its grants committees as well, says *Science*. While the organization can technically do as it sees fit, substantial political and financial pressure may be applied by the BMBF. The standoff between the DFG and the BMBF is the first of its kind between the two powerhouses, and the resolution promises to set a precedent. Science policy experts and scientists alike will be keeping an eye on this story. Making the U.S. Smithsonian Institution more efficient tops *Science*'s news items this week. *Nature* reports this story in second position. Smithsonian Secretary Lawrence Small, whose April budget proposal for the Smithsonian drew heavy fire from the scientific community, has revised his proposal to leave open the National Zoo's Conservation and Research Center (CRC). Still slated for closure is the Smithsonian's Center for Material Research and Education, whose work centers on the processing and preservation of anthropological artifacts. The CRC closure was first proposed for consideration of the property management burden it creates for the organization, says Small in *Science*. But the program provides breeding and research for many endangered species – providing valuable contributions to biological knowledge. The decision to keep the center open was made to correct the "misperception" that the Smithsonian is abandoning science, quotes *Nature*. A restructuring plan for the organization is in the planning stages, with many Smithsonian veterans feeling that Small's zoom-in on the sciences seems unfair, both journals report. This production-oriented approach to science funding seems to be a growing trend in the U.S., coming in the form of Smithsonian and National Science Foundation budget shrink-wrapping. Just how far will U.S. administrations go to undercut non-applied sciences? So far, it is a wait-and-see proposition. Also in the news of both *Nature* and *Science* is U.S. President George W. Bush's intention create a bigger, badder national missile defense system. Dubya has said publicly that he aims to protect the U.S. from smaller, nuclear-capable nations who regularly engage in terrorism. The Reagan-era dud reminder has already caused diplomatic ripples for the U.S., particularly with former Cold War opposition nation, Russia. Now scientists have their say. A meeting of the American Physical Society yielded speculation about the true risk of missile attacks on the U.S., and the feasibility of the incredibly expensive "layered" plan proposed by Bush. A panel of APS members plans to release a report on the project by the end of year to advise the president, as the society did to guide former U.S. president Reagan.
